# Exclusive breastfeeding practices and its determinants in Indian infants: findings from the National Family Health Surveys-4 and 5

**DOI:** 10.1186/s13006-023-00602-z

**Published:** 2023-12-20

**Authors:** Samarasimha Reddy N, Aravind Dharmaraj, Jovis Jacob, Kulandaipalayam Natarajan Sindhu

**Affiliations:** 1 Division of Clinical Epidemiology, ICMR – National Institute of Nutrition, Hyderabad, Telangana 500007 India; 2 The Wellcome Trust Research Laboratory, Division of Gastrointestinal Sciences, Christian Medical College, Vellore, Tamil Nadu 632004 India

**Keywords:** Exclusive breastfeeding, Factors, India, Infants, National Family Health Survey

## Abstract

**Background:**

The World Health Organization (WHO) recommends exclusive breastfeeding (EBF) in infants for the first 6 months of life. This analysis aims to estimate the proportion of Indian infants exclusively breastfed for the first 6 months using the National Family Health Surveys (NFHS)-4 and 5, and further, determine factors associated with EBF practices.

**Methods:**

EBF for this analysis was defined as when infants received only breast milk and no complementary feeds (solid food, water, animal milk, baby formula, juice, and fortified food) in the last 24 h prior to the survey. The proportion of infants exclusively breastfed was plotted from birth to 6 months as per the age of children at the time of the survey, and this was computed for individual states, union territories, and overall, for India. Univariate and multivariable logistic regression analyses were performed to examine factors influencing EBF in Indian infants.

**Results:**

The proportion of Indian infants exclusively breastfed for 6 months was 31.3% (1280/4095; 95% CI 29.9, 32.7) and 43% (1657/3853; 95% CI 41.4, 44.6) as per the NFHS-4 and 5 surveys, respectively. In NFHS-5, infants of scheduled tribes (aOR 1.5; 95% CI 1.2, 1.9) and mothers who delivered at public health facilities (aOR 1.3; 95% CI 1.1, 1.5) showed an increased odds of being exclusively breastfed at 6 months of life compared to their counterparts. Further, infants of mothers aged < 20 years (aOR 0.5; 95% CI 0.4, 0.7), low birth weight infants (aOR 0.6; 95% CI 0.4, 0.8), and infants in whom breastfeeding was initiated one hour after birth (aOR 0.8; 95% CI 0.7, 0.9) showed a reduced odds of being exclusively breastfed at 6 months compared to their counterparts.

**Conclusions:**

The overall EBF practice showed an increasing trend in the NFHS-5 compared to the NFHS-4 survey. However, a vast gap remains unaddressed in the Indian setting with > 50% of the population still not exclusively breastfeeding their infants for the WHO recommended duration of first 6 months. Behavioral studies dissecting the complex interplay of factors influencing EBF within the heterogenous Indian population can help plan interventions to promote and scale-up EBF in Indian infants.

**Supplementary Information:**

The online version contains supplementary material available at 10.1186/s13006-023-00602-z.

## Background

The World Health Organization (WHO) and United Nations International Children’s Emergency Fund (UNICEF) recommend exclusive breastfeeding (EBF) of infants for the first 6 months of life, and further to initiate complementary foods by the same time while continuing breastfeeding up to 2 years of age [1, 2]. EBF as defined by WHO implies that the infant is only on breast milk with no other liquids or solids, not even water, however with the exception of oral rehydration solution (ORS), vitamin syrup or drops, minerals, and medicines [1, 2].

Data from low- and middle-income countries (LMIC) between 2000 and 2019 among children younger than 2 years of age showed that the overall EBF rate increased to 48.6% (41.9–55.2) in 2019 [3]. The analysis also highlighted that EBF increased across all regions of the world except in the middle-east and north African regions [3]. Another analysis of data from 78 LMICs showed that the number of child deaths attributed to sub-optimal breastfeeding was 804,000, implying 11.6% of the total under-5 deaths in 2011 [4]. A pooled analysis from three prospective longitudinal cohorts in Ghana, India, and Tanzania showed that EBF for the first 6 months reduced morbidity and mortality among infants in the first 6 months, with low EBF practice reducing the overall child survival in the first 2 years of life [5].

Meta-analyses have shown that breastfeeding can markedly reduce mortality and morbidity attributed to infectious diseases such as diarrhea and pneumonia, this being critical in developing countries with a background of high infectious diseases burden [6–8]. From the maternal health perspective, multiple studies including systematic reviews and meta-analyses have provided evidence on the impact of EBF on maternal health, revealing that breastfeeding for more than 12 months was protective against breast and ovarian cancers, and played a role in preventing diabetes mellitus in the long run [9, 10]. In a review to identify the impact of breastfeeding on short- and long-term infant and maternal health outcomes in high-income settings, it was found that early cessation of breastfeeding or no breastfeeding at all was associated with an increased risk of maternal postpartum depression [11]. EBF has over time demonstrated multiple, holistic benefits for both the mother and baby, and it is thereby pivotal to understand and scale-up strategies to promote EBF for up to 6 months, and further, to promote continued breastfeeding for the first 2 years of life, especially in the LMIC settings [4, 11, 12].

In LMICs, only 37% of infants are exclusively breastfed for the recommended first six months of life [13]. As per the UNICEF, based on Multiple Indicator Cluster Surveys (MICS), Demographic and Health surveys (DHS) and other nationally representative databases (2015–2021), only 48% of the infants aged between 0 and 5 months are exclusively breastfed worldwide with the highest EBF prevalence of 61% being documented in the South Asian region [14]. The sub-Saharan region showed an EBF prevalence of 55% including eastern and southern Africa, 38% in western and central Africa, and 32% in middle-eastern and northern Africa [14]. As per the last three rounds of National Family Health Survey (NFHS) reports, EBF in Indian infants under 6 months of age increased from 46% during 2005-06 to 55% during 2015-16, and further to 65% during 2019-21 [15–17]. However, a prospective longitudinal birth cohort with intensive bi-weekly surveillance conducted in urban Vellore in southern India found that the EBF was less than 2% at 6 months of age [18]. Another study which studied pooled data from three longitudinal birth cohorts in south India, between 2002 and 2009, deduced the prevalence of exclusive breastfeeding for the first 6 months as 11.4% [19]. Cohort studies have intensive and multiple rounds of follow-ups, covering the entire first 6 months of infant’s life, thereby giving more accurate estimates on infants exclusively breastfed as compared to cross-sectional surveys [16–19].

India accounts for about one fifth of the world’s annual births, and is potentially a large market for commercial milk formulas (CMF) [20]. The CMF industry and marketing play a crucial role in EBF practice as CMF is advertised and promoted as a solution to parenting challenges, influencing mothers to formula feed their young infants [21]. Further, CMF advertising and marketing assert that these specialized feeding formulas help alleviate common issues in the infant such as crying, unsettled behaviour, bloating due to gas, and short durations of night sleep [21, 22]. The industry targets vulnerable mothers who self-report insufficient milk, through product endorsements, and when endorsed through healthcare professionals make mothers in believing that CMF is the best option [23]. In urban areas, CMF is readily available and seen as an easy option for mothers struggling with breastfeeding due to a strict or sometimes absent maternity leave policy [23]. Additionally, a lack of family support adds to the challenge in establishing and maintaining breastfeeding, forcing the mother to adopt CMF [24]. However, CMF is not only at a disadvantage for the infant but expensive, especially for low-income families, adding to the financial burden of the family [25].

The NFHS in India is a country-wide, multi-round, cross-sectional survey, and involves data collected from different age groups in a sample of households [16, 17]. The survey covers children aged between 0 and 6 months and are assessed for EBF at the time of the survey [16, 17]. It is important to note that children aged less than 6 months who were on EBF at the time of survey, does not necessarily imply that the child would have been continued on EBF until six months of age, given that there were no further follow-ups to capture this. The lack of this follow-up consequently can lead to a higher estimate on children being exclusively breastfed using the NFHS surveys compared to individual cohort studies where full follow-ups are performed. To overcome this, it is important that an estimate be systematically made on the proportion of children exclusively breastfed as per the data available for each month during the first 6 months of life.

It is critical to understand the socio-demographic and cultural factors that influence exclusive breastfeeding practices for the first 6 months of life in various settings. A systematic review and meta-analysis that summarized evidence from developed countries found that maternal employment, insufficient or lack of breast milk, associated maternal/infant morbidities, lactational difficulties, cultural norms, and maternal body image issues were the barriers associated with low practice of EBF up to 6 months of life [26]. Lack of support from family or the absence of social support systems was also identified as one of the barriers for continuing EBF for 6 months. Further, cultural beliefs such as giving water along with feeds (believed to aid in digestion), influence the sub-optimal practice of EBF for 6 months [26]. Understanding the intricate web of factors associated with EBF practice in the Indian setting will help planning targeted approaches for promoting and scaling up EBF for the recommended first six months. This secondary data analysis aimed to estimate the proportion of Indian infants exclusively breastfed for the first 6 months of life using the NFHS-4 and 5 survey datasets. Further, factors associated with continuing EBF at four, and thereon up to 6 months of age were studied.

## Methods

### Study setting, design and population

The NFHS survey is a nationally representative cross-sectional survey conducted by the Ministry of Health and Family Welfare (MoHFW), Government of India (GoI), and is coordinated by the International Institute of Population Sciences (IIPS), Mumbai. The nationwide NFHS survey collects household level data, that includes the under-five children, women, and men. In the NFHS-4 survey, 601,509 households in 640 districts, 29 states and 7 union territories in India were surveyed, with a response rate of 98%, and similarly in the NFHS-5 survey, 636,699 households in 707 districts, 28 states and 8 union territories were surveyed, with a response rate of 98%. A two-stage stratified sampling with villages and Census Enumeration Blocks (CEBs) as the primary sampling units (PSU) in the rural and urban areas, respectively, was adopted during the first stage. Within each PSU, the households were selected using systematic random sampling in the second stage. In both the NFHS surveys, all married eligible women at the time of the survey were interviewed. Information was obtained on demographics, socio-economic characteristics, antenatal care, postpartum care, breastfeeding duration and practices [27, 28]. Individual-level data from the NFHS-4 & 5 surveys were used for this analysis. We obtained permission from Demographic and Health survey (DHS) team to access the NFHS-4 (2015-16) and NFHS-5 (2019–2021) datasets. A detailed description of the NFHS survey methodology and sample size has been provided in the NFHS reports [27, 28].

### NFHS- 4 and 5 datasets

We accessed the ‘children recode file’ from the DHS program website [29]. We included infants aged between 0 and 6 months to study the proportion distribution of exclusive breastfeeding practices during the first 6 months across the NFHS-4 and 5 surveys. For estimating the EBF practices at each month of age, infants were categorized into age groups: 0–30 days (1 month), 31–60 days (2 months), 61–90 days (3 months), 91–120 days (4 months), 121–150 days (5 months) and 151–180 days (6 months).

### Exposure and outcome variables

The exposure variables used in this analysis were broadly divided into three categories: household, maternal and infant characteristics. The household characteristics included religion [Hindu, Muslim, and others (Christian, Sikh, Buddhist/neo-Buddhist, Jain, Jewish, Parsi/Zoroastrian, no religion)]; caste categorized as scheduled caste (SC), scheduled tribe (ST), other backward class (OBC) and others (do not belong to SC/ST/OBC); place of residence (urban/rural); and wealth index (calculated from a standard set of assets held by the household and classified as quintiles, with a quintile of 1–5 representing lowest, lower, middle, higher and highest classes, respectively) [27, 28].

The maternal characteristics included mother’s age in years; number of antenatal visits during the pregnancy for last birth (the ideal number of antenatal visits being at least 4); maternal education [no education, primary (1 to 5 years of schooling), secondary (6 to 12 years of schooling) and higher (> 12 years of schooling)]; type of delivery classified as normal vaginal and caesarean delivery; and place of delivery (home delivery, delivery at a government or private facility). Infant characteristics included gender (male or female); birth order; and birth weight (< 2000 g, 2000 to 2499 g and ≥ 2500 g).

In line with the WHO recommendation of exclusive breastfeeding that the infant should receive only breastmilk for 6 months of life with exception of oral rehydration solution, drops and syrups of vitamins, minerals and medicines, ‘exclusive breastfeeding’ for our analysis was defined as infants who were on only breast milk and not given any other complementary feeds (solid food, water, animal milk, baby formula, juice and fortified food) in the last 24 h prior to the survey [1]. We studied the factors associated with EBF among those who continued EBF for more than 4 months (> 120 days) and more than 5 months (> 150 days), respectively. For this, we compared children aged more than 120 days and still on EBF with those not on EBF by the time they reached 120 days of age. Similarly, we compared children aged more than 150 days and still on EBF with those not on EBF by the time they reached 150 days of age.

### Statistical analysis

Analysis was performed using descriptive statistics, univariate, and multivariable logistic regression. The proportion of infants exclusively breastfed was plotted according to age in months. The proportion of infants exclusively breastfed at 6 months of age (151–180 days) was calculated for the individual states as well as union territories, and for India overall. Further, descriptive statistics were performed to compute the proportion of infants exclusively breastfed for > 120 days and > 150 days of age, respectively by household, maternal and infant characteristics. Univariate and multivariable binary logistic regression were performed to examine the factors influencing EBF for > 120 and > 150 days of age. Univariate logistic regression was performed between the binary outcome variable i.e. exclusive breastfeeding (yes, no) and the independent study variables. Further, variables with *p*-value < 0.25 on univariate analysis were included in multivariable analysis. The multivariable logistic regression included religion, caste, rural or urban setting, wealth index, maternal age, maternal education, number of antenatal visits, mode of delivery, place of delivery, gender of the infant, birth weight and time of breastfeeding initiation following birth. Crude Odds Ratios (cOR) and adjusted Odds Ratio (aOR) with their 95% confidence interval (CI) are reported here. A *p*-value < 0.05 was considered statistically significant. All the descriptive statistics and analysis are presented here after adjusting for sampling weight, clustering, and strata. STATA version 14.1 (StataCorp LLC, College Station, TX, USA) was used for analysis and adjustment for sampling weight, clustering and strata was done using *svyset* command.

## Results

**Fig. 1:**
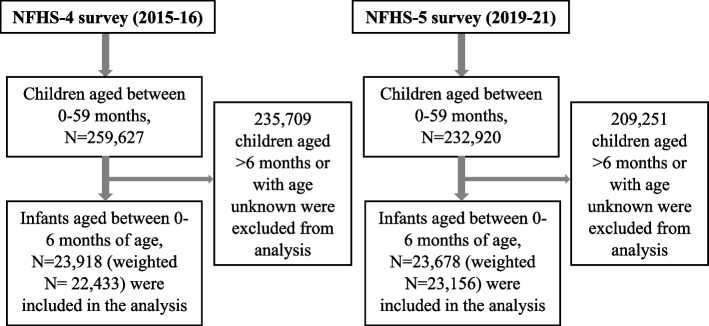
Flowchart depicting the process of data extraction from the NFHS-4 (2015-16) and 5 (2019-21) survey datasets

Of the 259,627 children aged under 5 years in the NHFS-4 survey, 23,918 infants aged between 0 and 6 months at the time of survey were included in the final analysis (weighted *N* = 22,433). Similarly, for the NHFS-5 survey, of the 232,920 under-five children, 23,678 infants aged between 0 and 6 months were included in the final analysis (weighted *N* = 23,156) (Fig. 1).

### Exclusive breastfeeding practices among Indian infants aged between 0 and 6 months

**Fig. 2:**
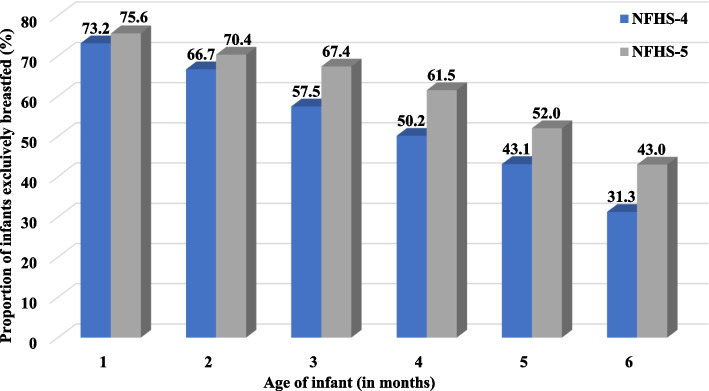
Proportion of Indian infants (0–6 months) exclusively breastfed as per the NFHS-4 (*N* = 22,433) and 5 (*N* = 23,156) surveys (weighted)

At 2 months of age, the proportion of infants exclusively breastfed was 66.7% (2268/3401; 95% CI 65.1, 68.3) during the NFHS-4 survey that increased to 70.4% (2770/3938; 95% CI 68.9, 71.8) during the NFHS-5 survey. At 4 months of age, the EBF proportion was 61.5% (2443/3972, 95% CI 60, 63) during the NFHS-5 survey which was higher when compared to the NFHS-4 survey at 50.2% (1958/3904; 95% CI 48.6, 51.7). At 6 months of age, the proportion of infants exclusively breastfed was again higher during the NFHS-5 survey with 43% (1657/3853; 95% CI 41.4, 44.6) infants being exclusively breastfed when compared to 31.3% (1280/4095; 95% CI 29.9, 32.7) during the NFHS-4 survey (Fig. 2 and Supplementary Table 1).

### Exclusive breastfeeding practices at 6 months among different states and union territories of India as per the NFHS-4 and NFHS-5 surveys

**Fig. 3:**
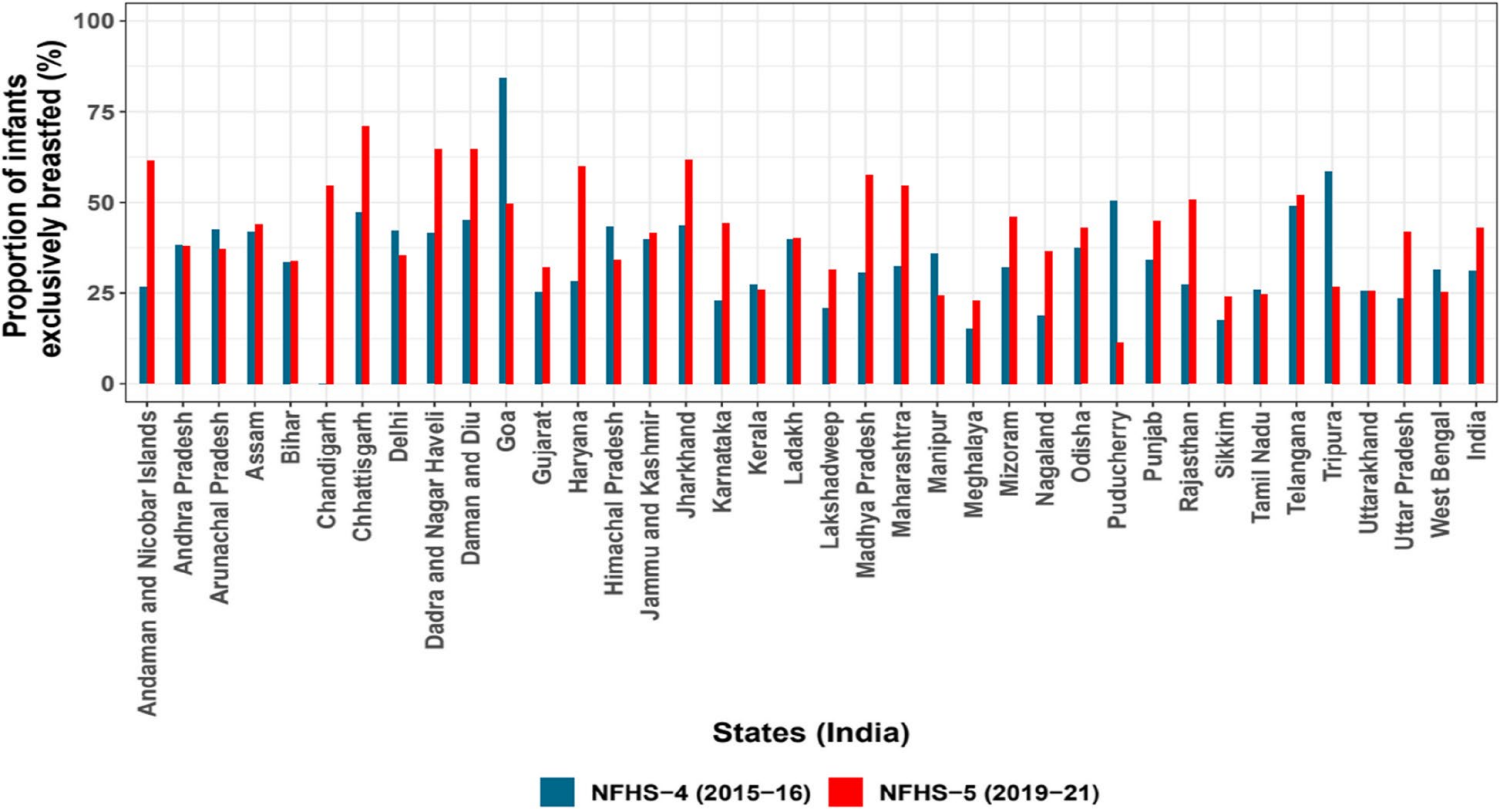
State-wise proportion of exclusive breastfeeding in infants upto 6 months (151–180 days) of age from NFHS-4 (*N* = 2889) and NFHS-5 (*N* = 3611) survey (weighted)

As per the NFHS-5 survey, EBF practices in Chhattisgarh (71%), Haryana (69.5%), and Jharkhand (61.7%) were higher when compared to the overall proportion of 43% for India, while the practice was lower in Meghalaya (23%), Manipur (24.5%), West Bengal (25.4%) and Uttarakhand (25.5%). In the NHFS-4 survey, EBF practices in Tripura (58.5%), Chhattisgarh (47.2%) and Himachal Pradesh (43.4%) were higher compared to the overall EBF practice of 31.3% for India, with the same being lower for Meghalaya (15.1%), Sikkim (17.5%), Karnataka (22.9%), and Uttar Pradesh (23.4%) (Fig. 3 and Supplementary Table 2).

## Exclusive breastfeeding practices among Indian infants at 6 months in relation to baseline characteristics

The baseline characteristics of exclusively breastfed infants for </> 120 days and </> 150 days are depicted in Table 1. EBF practices were higher among Hindu mothers with 20.7% exclusively breastfeeding their infants as per the NFHS-5 survey which increased from 14.6% during the NFHS-4 survey. The NFHS-5 survey showed that EBF practices among mothers with ≥ 4 antenatal visits during pregnancy was higher (21.8%) when compared to those with < 4 antenatal visits (18.9%) or no visits (14.9%). Further, the EBF practices as per the NFHS-5 survey among mothers who delivered at a public healthcare (government) facility was higher (22.2%) compared to those who delivered at a private healthcare facility (16.7%) or at home (14.5%). The EBF practices during the NFHS-5 survey were similar for both male (19.6%) and female (20%) infants whereas in the NFHS-4 survey it was higher among male (15.4%) when compared to female (12.6%) infants. EBF practices at 6 months were higher among mothers who breastfed immediately after birth (25.1%) when compared to those who breastfed within (22.6%) and after one hour of birth (18.1%) as per the NFHS-5 survey. EBF practices at 6 months were higher among children immediately breastfed after birth in NFHS-5 survey (50.3%) compared to NFHS-4 survey (25.1%) (Table 1).

**Table 1:** Socio-demographic, maternal and infant characteristics of those exclusively breastfeed for > 120 days and > 150 days (weighted) in the NFHS-4 *(N = 22,433)* and 5 *(N = 23,156)* surveys

Variable		Exclusive breastfeeding > 120 days		Exclusive breastfeeding > 150 days	
		NFHS-4 Yes (n, %)	NFHS-5 Yes (n, %)	NFHS-4 Yes (n, %)	NFHS-5 Yes (n, %)
***Household characteristics***					
**Religion**	Hindu	2415 (36.1)	2996 (44.8)	1037 (14.6)	1340 (20.7)
	Muslim	469 (31.6)	549 (37.8)	182 (11.1)	248 (16.7)
	Christian	54 (28.8)	79 (38.2)	21 (11.4)	33 (15.1)
	Others^a^	104 (45.7)	87 (45.1)	39 (17.1)	35 (18.3)
**Caste**	Scheduled caste	694 (37.6)	868 (42.9)	280 (14.8)	397 (20.2)
	Scheduled tribe	405 (42.6)	451 (51.4)	199 (19.7)	188 (24.2)
	Other backward class	1250 (33.3)	1617 (43.1)	504 (12.5)	721 (19.2)
	Others	565 (34)	615 (43.2)	256 (14)	256 (18.4)
**Setting**	Rural	2269 (36)	2825 (44.2)	993 (14.9)	1268 (20.4)
	Urban	773 (33.9)	887 (41.4)	287 (11.6)	389 (18)
**Wealth index (quintile)**	Highest	473 (35.1)	652 (47.5)	193 (13.4)	271 (21)
	Higher	553 (36.2)	595 (38.6)	220 (13.3)	256 (16.1)
	Middle	553 (32.6)	724 (45.1)	240 (13.1)	326 (20.4)
	Lower	678 (35.3)	822 (43.2)	268 (13.4)	402 (21.6)
	Lowest	784 (37.6)	918 (43.4)	359 (16.2)	401 (19.7)
***Maternal characteristics***					
**Maternal age (years)**	< 20	272 (39.9)	253 (40.8)	118 (17.1)	114 (18.3)
	20–24	1360 (36)	1544 (44.3)	542 (13.7)	700 (20.7)
	25–31	1167 (34.5)	1557 (44.2)	521 (14.1)	684 (19.7)
	> 31	243 (32.8)	358 (39.5)	100 (12.4)	158 (17.5)
**Maternal education**	Higher	357 (32.7)	712 (46.2)	147 (12.5)	292 (19.8)
	Secondary	1518 (36.6)	1930 (44)	601 (13.9)	880 (20.4)
	Primary	364 (33.1)	390 (40.7)	174 (14.1)	188 (19.9)
	No schooling	803 (35.9)	679 (41.2)	358 (14.9)	297 (18.1)
**Number of antenatal visits**	≥ 4	1586 (37.8)	2173 (45.9)	625 (14.2)	982 (21.8)
	< 4	993 (34.2)	1346 (43)	456 (14.5)	585 (18.9)
	No visits	439 (34)	164 (35.8)	188 (13.5)	72 (14.9)
**Mode of delivery**	Vaginal birth	2434 (35)	2822 (43.5)	1036 (14)	1257 (20)
	Caesarean section	608 (37.2)	889 (43.3)	244 (14.1)	400 (19.1)
**Place of delivery**	Public health facility	1759 (36.8)	2421 (45.9)	714 (14.3)	1124 (22.2)
	Private health facility	760 (33.3)	995 (40.9)	302 (12.1)	408 (16.7)
	Home delivery	516 (34.4)	292 (35.8)	262 (15.9)	124 (14.5)
***Infant characteristics***					
**Gender of infant**	Male	1607 (36.4)	1875 (42.5)	712 (15.4)	860 (19.6)
	Female	1434 (34.4)	1836 (44.6)	568 (12.6)	796 (20)
**Birth order**	1	1306 (42.2)	1577 (51.6)	543 (17.3)	718 (24.7)
	2	951 (31.3)	1162 (37.9)	389 (11.8)	504 (16.6)
	≥ 3	785 (32.1)	972 (40.4)	348 (12.8)	434 (17.9)
**Birth weight (g)**	≥ 2500	2121 (37.2)	2859 (46.7)	853 (14.3)	1263 (21.5)
	2000–2499	330 (33.5)	536 (40.3)	137 (12.8)	256 (19.4)
	< 2000	81 (29.6)	84 (27.1)	39 (12.7)	30 (8.5)
**Breastfeeding initiation after birth**	Immediately after birth	1386 (41)	1587 (50.3)	589 (17)	750 (25.1)
	< 1 h of birth	797 (38.6)	1276 (49)	359 (16)	549 (22.6)
	> 1 h of birth	859 (32.1)	848 (43)	333 (11.5)	357 (18.1)

^a^Others include Christian, Sikh, Buddhist/neo-Buddhist, Jain, Jewish, Parsi/Zoroastrian, no religion, and those not defined

### Factors affecting exclusive breastfeeding practices at 6 months among Indian infants as per NFHS-4 and 5 surveys

In the NFHS-5 survey, infants belonging to schedule caste (aOR 1.2; 95% CI 1, 1.5), schedule tribe (aOR 1.5; 95% CI 1.2, 1.9), or other backward classes (aOR 1.3; 95% CI 1.1, 1.5) were at an increased odds of being exclusively breastfed for 6 months compared to those belonging to other categories. In the NFHS-5 survey, infants belonging to Muslim (aOR 0.8; 95% CI 0.6, 0.9) or Christian religion (aOR 0.5; 95% CI 0.4, 0.6) had a decreased odds of being exclusively breastfed for 6 months compared to those from Hindu religion. Infants of mothers aged < 20 years (aOR 0.5; 95% CI 0.4, 0.7) were at a decreased odds of being exclusively breastfed at 6 months compared to mothers aged between 25 and 31 years. Also, the NFHS-5 survey showed that infants whose mothers delivered at a public (government) healthcare facility (aOR 1.3; 95% CI 1.1, 1.5) were more likely to exclusively breastfeed their infants for 6 months compared those mothers who delivered at a private healthcare facility. Low birth weight infants (< 2000 g) (aOR 0.6; 95% CI 0.4, 0.8) and infants with birth order ≥ 3 (aOR 0.7; 95% CI 0.6, 0.8) were at a decreased odds of being exclusively breastfed for 6 months compared to their counterparts. Further, infants of mothers who breastfed after one hour of birth (aOR 0.8; 95% CI 0.7, 0.9) were at decreased odds of continuing EBF for upto 6 months when compared to those who were breastfed immediately after birth. In the NFHS-4 survey, infants from rural areas (aOR 1.3; 95% CI 1.1, 1.6), showed an increased odds of being exclusively breastfed for 6 months compared to those from urban areas whereas no such difference was noted in the NFHS-5 survey (Table 2).

**Table 2:** Factors associated with exclusive breastfeeding for > 120 and > 150 days in the NFHS-4 and 5 surveys (weighted) *(> 120 days - NFHS-4, N = 6324; NFHS-5, N = 6890 & > 150 days - NFHS-4, N = 6557; NFHS-5, N = 6334)* using multivariable logistic regression analysis

Variable		Exclusive breastfeeding > 120 days				Exclusive breastfeeding > 150 days			
		NFHS-4 aOR [95% CI]	*P*-value	NFHS-5 aOR [95% CI]	*P*-value	NFHS-4 aOR [95% CI]	*P*-value	NFHS-5 aOR [95% CI]	*p*-value
***Household characteristics***									
**Religion**	Hindu	Ref		Ref	Ref	Ref	Ref	Ref	Ref
	Muslim	0.8 [0.7, 1]	0.023	0.8 [0.7, 0.9]	0.002	0.8 [0.6, 1]	0.023	0.8 [0.6, 0.9]	0.008
	Christian	0.6 [0.5, 0.8]	0.000	0.5 [0.4, 0.6]	0.000	0.6 [0.4, 0.8]	0.001	0.5 [0.4, 0.6]	0.000
	Others	1.1 [0.9, 1.4]	0.423	0.7 [0.6, 0.9]	0.006	1 [0.7, 1.5]	0.829	0.8 [0.6, 1.1]	0.099
**Caste**	Schedule caste	0.9 [0.8, 1.1]	0.309	1.1 [1, 1.3]	0.164	0.8 [0.7, 1.1]	0.145	1.2 [1, 1.5]	0.037
	Schedule tribe	1.4 [1.1, 1.6]	0.002	1.4 [1.2, 1.7]	0.000	1.3 [1.1, 1.7]	0.019	1.5 [1.2, 1.9]	0.001
	Other backward class	0.9 [0.8, 1.1]	0.300	1.2 [1, 1.4]	0.015	0.9 [0.7, 1]	0.098	1.3 [1.1, 1.6]	0.009
	Others	Ref	Ref	Ref	Ref	Ref	Ref	Ref	Ref
**Setting**	Rural	1.1 [1.0, 1.3]	0.168	1.1 [0.9, 1.2]	0.494	1.3 [1.1, 1.6]	0.008	1.2 [1, 1.4]	0.116
	Urban	Ref	Ref	Ref	Ref	Ref	Ref	Ref	Ref
**Wealth index (quintile)**	Highest	Ref	Ref	Ref	Ref	Ref	Ref	Ref	Ref
	Higher	0.9 [0.8, 1.1]	0.517	0.8 [0.6, 0.9]	0.004	0.9 [0.7, 1.2]	0.505	0.8 [0.7, 1]	0.080
	Middle	0.9 [0.7, 1]	0.124	0.9 [0.8, 1.1]	0.521	0.9 [0.7, 1.1]	0.227	1 [0.8, 1.2]	0.930
	Lower	1 [0.8, 1.2]	0.749	0.9 [0.7, 1.1]	0.238	0.9 [0.7, 1.1]	0.270	1 [0.7, 1.2]	0.659
	Lowest	1.1 [0.9, 1.4]	0.358	1 [0.8, 1.2]	0.922	1 [0.8, 1.4]	0.857	1 [0.8, 1.3]	0.873
***Maternal characteristics***									
**Maternal age (years)**	< 20	0.9 [0.7, 1.1]	0.196	0.6 [0.5, 0.7]	0.000	0.8 [0.6, 1.1]	0.138	0.5 [0.4, 0.7]	0.000
	20–24	0.9 [0.8, 1.1]	0.319	0.9 [0.8, 1]	0.021	0.9 [0.7, 1]	0.110	0.8 [0.7, 1]	0.015
	25–31	Ref	Ref	Ref	Ref	Ref	Ref	Ref	Ref
	> 31	1.1 [0.9, 1.4]	0.199	1.1 [0.9, 1.3]	0.406	1 [0.8, 1.3]	0.945	1 [0.8, 1.3]	0.833
**Maternal education**	Ref	Ref	Ref	Ref	Ref	Ref	Ref	Ref	Ref
	Secondary	1.1 [0.9, 1.1]	0.441	1 [0.9, 1.2]	0.626	1.1 [0.8, 1.3]	0.636	1.1 [0.9, 1.3]	0.493
	Primary	0.9 [0.7, 1.1]	0.405	1 [0.8, 1.3]	0.855	0.9 [0.6, 1.2]	0.443	1.1 [0.9, 1.5]	0.284
	No education	1 [0.8, 1.2]	0.884	1 [0.8, 1.2]	0.977	1 [0.7, 1.3]	0.751	1 [0.8, 1.3]	0.866
**Number of antenatal visits**	≥ 4 visits	Ref	Ref	Ref	Ref	Ref	Ref	Ref	Ref
	< 4 visits	0.9 [0.8, 1]	0.039	1 [0.9, 1.2]	0.535	1.1 [0.9, 1.2]	0.459	1 [0.8, 1.1]	0.412
	No visits	1 [0.8, 1.2]	0.697	0.9 [0.7, 1.2]	0.403	0.9 [0.7, 1.2]	0.636	0.9 [0.7, 1.3]	0.605
**Mode of delivery**	Vaginal birth	Ref	Ref	Ref	Ref	Ref	Ref	Ref	Ref
	Caesarean section	1.1 [1, 1.3]	0.108	1 [0.9, 1.2]	0.560	1.1 [1, 1.4]	0.093	1 [0.9, 1.2]	0.897
**Place of delivery**	Public health facility	1.2 [1, 1.4]	0.017	1.1 [1, 1.3]	0.087	1.2 [1, 1.4]	0.106	1.3 [1.1, 1.5]	0.003
	Private health facility	Ref	Ref	Ref	Ref	Ref	Ref	Ref	Ref
	Home delivery	1.2 [0.9, 1.5]	0.252	1 [0.8, 1.2]	0.765	1.3 [0.9, 1.7]	0.162	19 [0.7, 1.3]	0.939
***Infant characteristics***									
**Gender of infant**	Male	Ref	Ref	Ref	Ref	Ref	Ref	Ref	Ref
	Female	1 [0.9, 1.1]	0.917	1.1 [1, 1.2]	0.121	1 [0.8, 1.1]	0.526	1.1 [0.9, 1.2]	0.350
**Birth order**	1	Ref	Ref	Ref	Ref	Ref	Ref	Ref	Ref
	2	0.6 [0.5, 0.7]	0.000	0.5 [0.4, 0.5]	0.000	0.6 [0.5, 0.7]	0.000	0.5 [0.4, 0.6]	0.000
	≥ 3	0.6 [0.5, 0.7]	0.000	0.5 [0.4, 0.7]	0.000	0.7 [0.5, 0.8]	0.000	0.5 [0.4, 0.6]	0.000
**Birth weight (g)**	≥ 2500	Ref	Ref	Ref	Ref	Ref	Ref	Ref	Ref
	2000–2499	0.8 [0.7, 0.9]	0.003	0.8 [0.7, 1]	0.013	0.9 [0.7, 1.1]	0.237	1 [0.8, 1.1]	0.569
	< 2000	0.9 [0.7, 1.2]	0.473	0.7 [0.5, 0.9]	0.007	1 [0.7, 1.4]	0.802	0.6 [0.4, 0.8]	0.003
**Breastfeeding initiation after birth**	Immediately after birth	Ref	Ref	Ref	Ref	Ref	Ref	Ref	Ref
	< 1 h of birth	0.9 [0.8]	0.052	1 [0.9, 1.1]	0.845	1 [0.9, 1.2]	0.941	1 [0.9, 1.2]	0.997
	> 1 h of birth	0.68 [0.6, 0.8]	0.000	0.7 [0.6, 0.8]	0.000	0.6 [0.5, 0.7]	0.000	0.8 [0.7, 0.9]	0.001

*Abbreviation:* *aOR* Adjusted odds ratio

## Discussion

The present analysis that was performed to understand exclusive breastfeeding practices among Indian mothers using the nationally representative NFHS-4 and 5 surveys. The proportion of Indian infants exclusively breastfed for 6 months showed an increase from 31.3% during the NFHS-4 (2015-16) survey to 43% in the NFHS-5 (2019–2021) survey as per the definitions used in this analysis. The mothers from the scheduled tribe community, delivering at public health facilities, and having had ≥ 4 antenatal visits during pregnancy showed an increased odds of exclusively breastfeeding their infants for upto 6 months. Younger mothers (< 24 years), low birth weight infants (< 2000 g), higher birth order (≥ 3), and initiation of breastfeeding more than one hour following birth were associated with a reduced odds of being exclusively breastfed for upto 6 months of life. Place of residence (rural or urban), wealth index and infant gender played no role in influencing exclusive breastfeeding for 6 months.

### Comparison of exclusive breastfeeding practices in Indian infants with other settings

Our analysis showed that 31.3% and 43% of Indian infants from the NFHS-4 and 5 surveys, respectively, were exclusively breastfed for up to 6 months of age (151–180 days) which is low when compared to the reported national prevalence for India of 54.9% and 63.7%, respectively (as per the NFHS-4 and 5) survey reports [16, 17]. The reasons for this lower prevalence estimates in our analysis can be explained by the fact that for estimating EBF practices at 6 months of age, we included only age-appropriate children, that is children aged between 151 and 180 days at the time of the survey.

Our analysis estimates are lower than estimates from other cross-sectional studies conducted across India that have reported EBF percentages for up to 6 months as ~ 50% [30, 31]. Our analysis showed that EBF was 61.5% and 43% at four and 6 months of age, respectively (NFHS-5 survey). This finding is similar to the study from the neighboring country of Sri Lanka where EBF at 4 months was higher (62%) when compared to 6 months (16%) [32]. It is to be reiterated that NFHS surveys are based on maternal recall. In a study by Andarge et al. which assessed recall period accuracies found that one week recall period gave a more accurate estimate of exclusive breastfeeding practice than a 24-hour recall among infants younger than 6 months of age. This study by Andarge et al. found that the EBF was estimated at 71% from a single 24-hour recall, an overestimate when compared to 47% EBF computed from repeated weekly surveillance [33]. Given that the NFHS surveys for breastfeeding are based on a 24-hour recall, EBF at six months of age in our analysis was estimated at 43% which is very high compared to EBF of 2% from a longitudinal birth cohort study with bi-weekly surveillance [18].

### Factors affecting exclusive breastfeeding practices at six months of age in Indian infants

The present analysis showed that the differences in EBF practices based on the infant’s gender at 6 months of age was statistically significant in the NFHS-4 data but did not hold true for the NFHS-5 data. This result is consistent with an urban cohort study conducted in Vellore, southern India [18, 19]. This is a notable and encouraging finding that gender disparities that existed in the past may have narrowed now in the Indian setting. EBF practice among scheduled tribes is known to be high, and this is substantiated by the present analysis [34]. The majority of scheduled tribes belong to low socioeconomic background, and the reason for the high rate of exclusive breastfeeding in this population could be attributed to the reduced financial access to breastfeeding substitutes or formula foods [34]. It is interesting to note that EBF practices as per the NFHS-5 survey were significantly higher among mothers who delivered at public healthcare facilities when compared to those who delivered at private healthcare facilities or at home. This clearly indicates an improved and expanded access to maternal/breastfeeding counselling and care at public health facilities that are providing early education and motivation to mothers, perhaps becoming pivotal in sustaining continued EBF for up to 6 months of age [35, 36]. Also, there is a possibility that mothers who deliver at public health facilities cannot afford breastmilk substitutes compared to those delivering at private health facilities. The NFHS-5 data further has highlighted that a higher number of antenatal visits during pregnancy (≥ 4) implied a greater chance of the mothers going on to exclusively breastfeed their infants for up to 6 months suggesting that healthcare workers (anganwadi workers/peripheral health nurses/doctors) during antenatal visits and follow-ups are playing a key role in educating and encouraging the mothers-to-be about the benefits of EBF along with support through other maternal healthcare programs and postnatal care facilities provided by the government [36–38].

Young mothers (< 24 years) seemed to less likely breastfeed their infants exclusively up to 6 months. This can perhaps be attributed to the lack of awareness about the benefits and misconceptions regarding EBF [39–41]. The same was seen with infants born with lower birth weight perhaps explained by the fact that these are high-risk infants requiring nursery care and support, delaying the initiation and continuation of EBF, and possibly put on formula feeds during and after nursery care [39–41]. According to the NFHS-5 data, the higher the birth order, lower was the EBF practice for up to 6 months. This can possibly be explained by the fact that an increased family size demands the mother getting back to household chores early or work to avoid loss of wages, and thereby being unable to exclusively breastfeed her infant for the recommended 6 months due to greater and conflicting demands.

The Infant Milk Substitutes, Feeding Bottles and Infant Foods (Regulation of Production, Supply and Distribution) act, 1992 as amended in 2003 (IMS act) aligns with the objectives of the International Code of Marketing of Breastmilk Substitutes [42]. The IMS act has played a crucial role in India in controlling the promotion and advertisement of CMFs. The act prohibits pregnant women or mothers being contacted directly for the purpose of promoting infant milk substitutes or infant foods [43]. Additionally, it provides recommendations for healthcare workers on how measures could be taken to prevent being influenced by baby food manufacturing companies [43]. Notably, India is one of the few countries in Asia to implement the guideline fully and has ensured that breastfeeding is strictly not undermined by the marketing of breastmilk substitutes [44]. In addition to protecting breastfeeding practices from being influenced or impacted by commercial promotion of CMFs, the IMS Act also regulates the marketing practices of baby food manufacturers [43]. Also, the implementation of the IMS Act by the Breastfeeding Promotion Network of India (BPNI) in 49 districts has helped improving EBF rates [45]. During the last 10 years (2010–2020), breastfeeding in India has increased by 5% as a result of the IMS Act, Maternity Benefits Act (adopted in 2017 to extend paid maternity leave for upto 26 weeks), and the government’s ‘Mother’s Absolute Affection’ campaign that promotes breastfeeding through counselling [46].

### Strengths, limitations and recommendations

This analysis that used the extensive, nationally representative NFHS-4 and 5 survey datasets covering all states and union territories of India, allowed the comparison of changes over time in EBF practices, and further to deduce factors influencing EBF, and this is the major strength of the study. In this study only eligible children who belonged to appropriate age groups were considered for analyzing the prevalence of exclusive breast feeding. However, a few limitations must be considered when interpreting the results of this analysis. In cross sectional surveys like these, estimates of EBF are based on maternal recall and this recall bias could potentially overestimate the prevalence of EBF practices reported. Despite these inherent limitations, there is overwhelming evidence that the NFHS surveys have provided valuable information on key population and health issues and have been instrumental in building India’s decision-making capacity and policies on health [17]. It is to be noted that NFHS data are collected by intensively trained staff, and the NFHS-4 and 5 surveys have a high response rate [17].

Qualitative studies to understand the driving factors among populations that have shown high exclusive breastfeeding rates such as among mothers from scheduled tribes and those who delivered at public healthcare facilities will help throw light to scale up the same amongst mothers of low birthweight babies in other Indian settings and backgrounds. Interventions and support targeted towards young mothers, mothers with low birth weight babies, and strongly encouraging mothers to initiate breastfeeding immediately after birth are some of the premises to be worked upon to enhance exclusive breastfeeding rates for up to 6 months in the Indian setting.

## Conclusion

Our analysis found that exclusive breastfeeding practices in the Indian setting was higher in the NFHS-5 survey when compared to NFHS-4 survey from birth to 6 months. The practices of EBF at 6 months was seen to be better among mothers from scheduled tribes, those who delivered at public healthcare facilities and those with adequate number of antenatal visits during pregnancy. EBF for 6 months was low among young mothers, those with low-birth-weight infants, higher birth order, and mothers who initiated breastfeeding after one hour of birth. An enhanced focus on promoting EBF practices by targeting special groups identified from this analysis through the existing maternal and child national health programmes such as Janani shishu suraksha Karyakram (JSSK), home-based newborn care (HBNC), and home-based care of young child (HBYC) must be meticulously harnessed to improve exclusive breastfeeding rates for up to 6 months. Added to this, qualitative studies to understand the intricate and complex driving factors associated with exclusive breastfeeding practices in the Indian setting could be pivotal in scaling up the same across all settings in India.

### Supplementary Information

**Additional file 1.**

## Data Availability

Data sharing is not applicable to this article as no datasets were generated or analysed during the current study. The NFHS-4 and 5 datasets are available on the DHS platform: https://dhsprogram.com/data/.

## Abbreviations

aOR: Adjusted Odds Ratio
cOR: Crude Odds Ratio
CI: Confidence Interval
CMF: Commercial Milk Formula
DHS: Demographic and Health surveys
EBF: Exclusive Breastfeeding
GOI: Government of India
IIPS: International Institute of Population Sciences
LMIC: Low–and Middle–income countries
MICS: Multiple Indicator Cluster Surveys
NFHS: National Family Health Survey
PHC: Primary Health Centre
WHO: World Health Organization
