# Supplementary material for: Exclusive breastfeeding practices and its determinants in Indian infants: findings from the National Family Health Surveys-4 and 5
Source: Int Breastfeed J. 2023 Dec 20;18:69. doi: 10.1186/s13006-023-00602-z (PMC10731841; doi:10.1186/s13006-023-00602-z)
Supplement: Supplementary file 1 — Additional file 1. [file 13006_2023_602_MOESM1_ESM.docx]

**Supplementary Table 1: Proportion of Indian infants (0-6 months) exclusively breastfed as per the NFHS-4 *(N=22,433)* and 5 surveys *(N=23,156)***

| **Age (months)** | **NFHS-4^a^** | | | **NFHS-5^a^** | | |
| --- | --- | --- | --- | --- | --- | --- |
|  | ***N*** | **EBF *(n)*** | **EBF % (95% CI)** | ***N*** | **EBF *(n)*** | **EBF % (95% CI)** |
| 1 (0-30 days) | 3155 | 2308 | 73.16 [71.58, 74.67] | 3609 | 2729 | 75.61 [74.18, 76.98] |
| 2 (31-60 days) | 3401 | 2268 | 66.69 [65.08, 68.25] | 3938 | 2770 | 70.35 [68.90, 71.75] |
| 3 (61-90 days) | 3792 | 2178 | 57.45 [55.87, 59.01] | 3835 | 2585 | 67.40 [65.90, 68.86] |
| 4 (91-120 days) | 3904 | 1958 | 50.15 [48.58, 51.71] | 3972 | 2443 | 61.51 [59.98, 63.00] |
| 5 (121-150 days) | 4086 | 1762 | 43.11 [41.59, 44.63] | 3949 | 2055 | 52.04 [50.47, 53.59] |
| 6 (151-180 days) | 4095 | 1280 | 31.26 [29.85, 32.69] | 3853 | 1657 | 42.99 [41.43, 44.55] |
| **Total** | **22,433** | **11,754** | **52.40 [51.74, 53.05]** | **23,156** | **14,239** | **61.50 [60.86, 62.11]** |

*^a^Weighted*
